# Ketogenic Diet for Obesity: Friend or Foe?

**DOI:** 10.3390/ijerph110202092

**Published:** 2014-02-19

**Authors:** Antonio Paoli

**Affiliations:** Department of Biomedical Sciences, University of Padova, Padova 35131, Italy; E-Mail: antonio.paoli@unipd.it; Tel.: +39-049-8275318; Fax: +39-049-8275301

**Keywords:** ketogenic diet, obesity, metabolism, ketone bodies

## Abstract

Obesity is reaching epidemic proportions and is a strong risk factor for a number of cardiovascular and metabolic disorders such as hypertension, type 2 diabetes, dyslipidemia, atherosclerosis, and also certain types of cancers. Despite the constant recommendations of health care organizations regarding the importance of weight control, this goal often fails. Genetic predisposition in combination with inactive lifestyles and high caloric intake leads to excessive weight gain. Even though there may be agreement about the concept that lifestyle changes affecting dietary habits and physical activity are essential to promote weight loss and weight control, the ideal amount and type of exercise and also the ideal diet are still under debate. For many years, nutritional intervention studies have been focused on reducing dietary fat with little positive results over the long-term. One of the most studied strategies in the recent years for weight loss is the ketogenic diet. Many studies have shown that this kind of nutritional approach has a solid physiological and biochemical basis and is able to induce effective weight loss along with improvement in several cardiovascular risk parameters. This review discusses the physiological basis of ketogenic diets and the rationale for their use in obesity, discussing the strengths and the weaknesses of these diets together with cautions that should be used in obese patients.

## 1. Introduction

Obesity is a rapidly growing epidemic worldwide [1] that has nearly doubled since 1980. In 2008, over 200 million men and nearly 300 million women aged 20 and over were obese, and 65% of the world’s population live in countries where overweight and obesity kills more people than underweight [2]. For family physicians, obesity is one of the most challenging problems confronted in daily practice and despite the efforts of both patients and physicians, this disorder is increasing in prevalence. Obesity is one of the principle risk factors for cardiovascular disease and along with dyslipidaemia, hypertension and diabetes contributes to the metabolic syndrome [3]. Many strategies have been proposed for reducing energy intake (diets, drugs, and bariatric surgery) [4] and for increasing energy output (exercise and non-exercise movement) [5], but even though there may exist a general agreement about the fundamental conceptual basis—changing energy intake and physical activity levels— how to achieve these goals is less clear. Regarding obesity interventions, diet is one of the more controversial issues and many different types have been advocated for weight loss, but there is little scientific evidence to recommend one diet over another. As a matter of fact there are still no definitive data on what dietary protocols are most effective in both the short and long term [6], or even what is the correct nutritional approach in general [7]. The most commonly accepted dietary strategy is based on relatively high levels of carbohydrates and low fat content, but according to some studies these low fat diets yield only modest weight losses and suffer from low long-term compliance issues [8]. In fact adherence of obese individuals to high carbohydrate/low fat nutrition is often a problem because the majority have been shown to have dietary preferences for foods with a rich fat content [9,10]. Another problem is that, in general, obese individuals prefer highly processed foods containing simple sugars rather than complex/raw carbohydrates; thus a low fat diet could actually encourage the consumption of sugars and refined carbohydrates that can worsen weight problems and also facilitate dyslipidemia, especially in insulin resistance individuals [11]. As a consequence of the debatable efficacy of these types of diet, there has been increased interest in recent years in very low carbohydrate ketogenic diets (VLCKDs) or simply ketogenic diets (KDs).

## 2. Ketogenic Diets in the Clinic

Even though the ketogenic diet may be useful as part of the treatment of various diseases (see Paoli *et al*. [11]), especially in pediatric pharmacoresistant epilepsy [12], the more common situation for the general practitioner is the use of KD by patients in order to lose weight. KDs have undoubtedly been shown to be effective, at least in the short to medium term, as a tool to fight obesity [13], hyperlipidemia and some cardiovascular risk factors [14,15,16], but ketogenic diets also raise some concerns among physicians [17]. Many of the concerns about the use of ketogenic diet as therapeutic tools could be attributed to a broad lack of knowledge about the physiological mechanisms involved. Ketogenic diets induce a metabolic condition named “physiological ketosis” by Hans Krebs, to distinguish it from the pathological diabetic ketosis [18]. 

## 3. The Physiology of Ketosis

After a few days of fasting or a drastically reduced carbohydrate diet (below 20 g per day), the body’s glucose reserves become insufficient for the production of oxaloacetate for normal fat oxidation in the Krebs cycle and for the supply of glucose to the central nervous system (CNS) [19,20,21,22]. 

Regarding the first issue, oxaloacetate is relatively unstable at body temperature, thus it is necessary (a minimal amount of oxaloacetate is required for an optimal functioning of the Krebs cycle) to supply the tricarboxylic acid cycle with oxaloacetate derived from glucose through ATP dependent carboxylation of pyruvic acid by pyruvate carboxylase [23]. 

**Figure 1:**
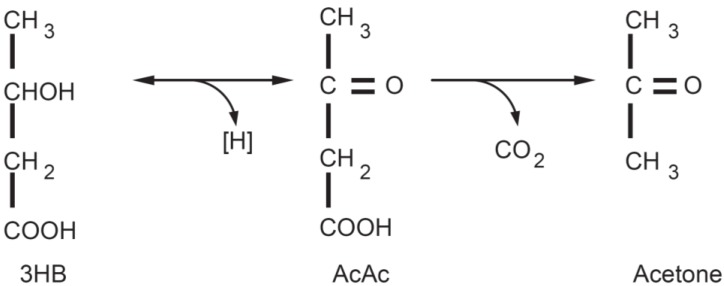
Ketone bodies: acetoacetate (AcAc) is the principle ketone body. It is produced and utilized during intermediary metabolism and other ketone bodies are derived from it. Acetone is produced by the spontaneous decarboxylation of acetoacetate and is important from the clinical point of view because it is responsible for the fruity sweet odour of infant ketoacidosis. β-Hydroxybutyric acid is produced via the reduction of AcAc. From a strictly biochemical point of view it is not actually a ketone body since the ketonic moiety is reduced to a hydroxyl group; it is though grouped among the ketone bodies. 3HB is relatively stable biochemically and is transported to the tissues where it is reconverted to AcAc.

**Figure 2:**
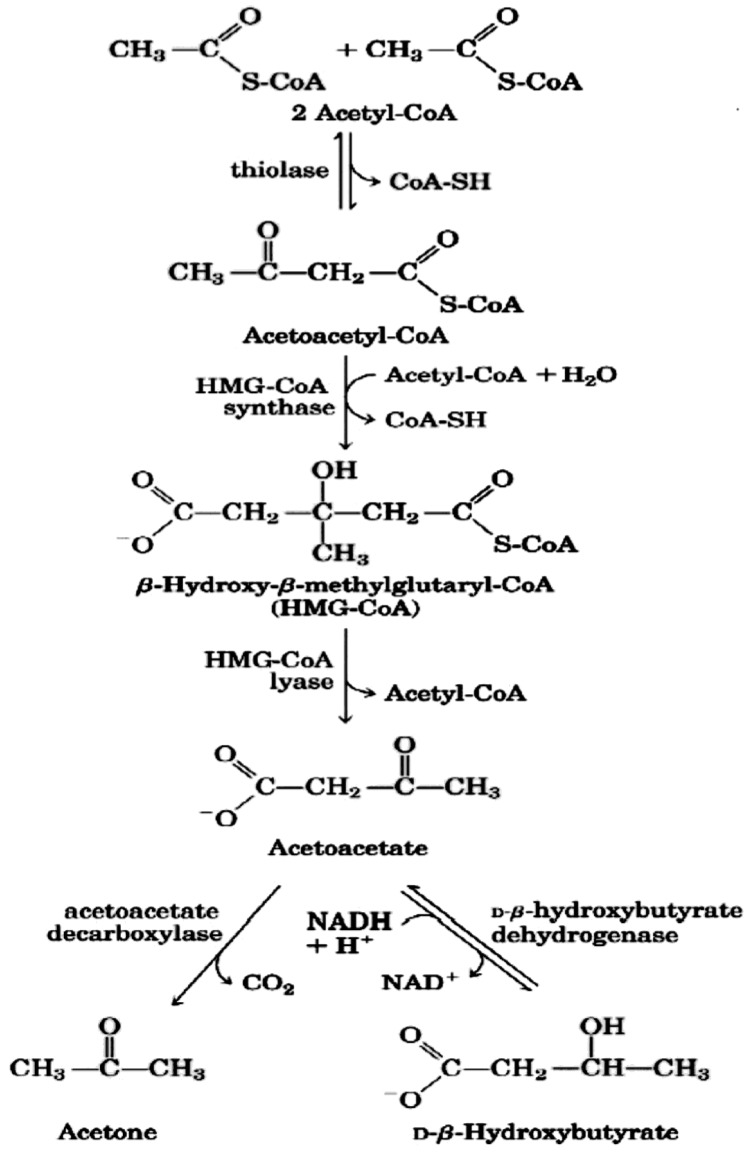
Pathway of ketone bodies’ formation from acetyl-CoA.

Regarding the second issue, the CNS cannot use fatty acids as an energy source (because they do not cross the blood-brain barrier), thus glucose is ordinarily the sole fuel for the human brain [24]. After 3–4 days of fasting or a very low carbohydrate diet the CNS needs an alternative energy source [19,20,21,22] and this is derived from the overproduction of acetyl-CoA which leads to the production of so-called ketone bodies (KB): acetoacetate (AcAc), β-hydroxybutyric acid (BHB) and acetone (Figure 1 and Figure 2). This process is called ketogenesis and occurs principally in the mitochondrial matrix in the liver [25]. It is important to underline that the liver produces ketone bodies, but is unable to utilize them because the absence of the enzyme 3-ketoacyl CoA transferase required to convert acetoacetate into acetoacetyl-CoA. 

Even though the main ketone body produced in the liver is acetoacetate, the primary circulating ketone is β-hydroxybutyrate that is not, strictly speaking, a ketone body because the ketone moiety has been reduced to a hydroxyl group. Under normal conditions the production of free acetoacetic acid is negligible and this compound, transported via the blood stream, is easily metabolised by various tissues, especially skeletal muscle and the heart. In conditions of overproduction, acetoacetic acid accumulates above normal levels and part is converted to the other two ketone bodies. The presence of ketone bodies in the blood and their elimination via urine causes ketonemia and ketonuria. Acetone (produced by spontaneous decarboxylation of acetoacetate), being a very volatile compound, is eliminated mainly via respiration in the lungs (hence the characteristic breath odour, the classic “fruity breath”) and, even though it does not have metabolic functions, its presence can be useful from a clinical diagnostic point of view. Thus it is to be considered that a “fruity breath” indicates a condition of ketosis that could be physiological (fasting, low carbohydrate diet, post exercise) not necessarily an index of a pathological condition. It is of interest to underline that ketosis is a metabolic state characteristic of humans, even if not unique, for example, due the relatively high-fat and low carbohydrate content in rodent milk the concentration of ketone bodies in suckling offspring can ranges between 1 and 2 mmol and this influences the increase in active uptake of BHB by the brain. However humans are more susceptible to ketosis-induced fasting due to the greater brain/body mass (this can explain why newborns are more susceptible to ketosis) [26]. Under normal conditions the concentration of ketone bodies is very low (<0.3 mmol/L) compared to glucose (approx. 4 mmol) and, since glucose and ketone bodies have a similar K_m_ (or Michaelis-Menten constant) for glucose transport to the brain, the ketone bodies begin to be utilised as an energy source by the CNS when they reach a concentration of about 4 mmol/L [27] which is close to the K_m_ for the monocarboxylate transporter [28]. 

**Figure 3:**
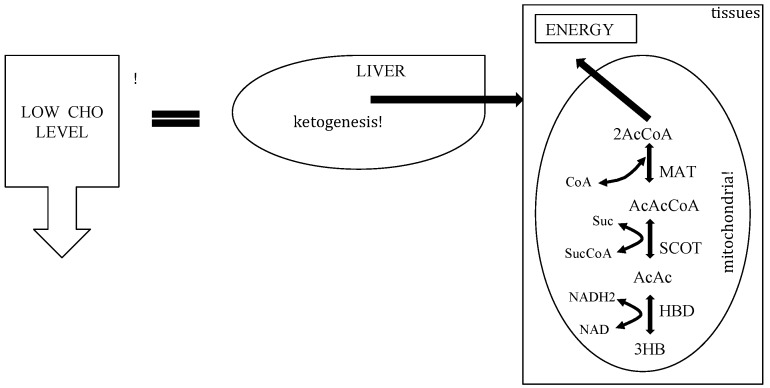
Metabolic pathway of ketosis and tissues ketolysis.

Ketone bodies are then used by tissues as a source of energy [25,27,29,30] through a pathway that involves firstly that BHB is converted back to AcAc this is then transformed into acetoacetyl-CoA and, finally, two molecules of acetyl-CoA are formed from acetoacetyl-CoA which are used in the Krebs cycle (Figure 3). It should be underlined that, as shown in Table 1, glycaemia, even though reduced, remains within physiological levels [31]. In fact glucose is formed from two sources: glucogenic amino acids and from glycerol liberated via lysis from triglycerides [32,33]. The importance of the second source increases progressively during the ketosis condition. In the first days of a ketogenic diet the main source of glucose is via neoglucogenesis from amino acids (AA), as the days goes by, the contribution of AA decreases whilst the amount of glucose derived from glycerol increases. As a matter of fact glycerol (released from triglyceride hydrolysis) can produce more than 16% of glucose in the liver during a KD and about 60% after a few days of complete fasting [32]. According to Bortz (1972) of the new glucose formed from protein and glycerol 38% is derived from glycerol in the lean and 79% in the obese [34]. 

**Table 1:** Blood levels during a normal diet, ketogenic diet and diabetic ketoacidosis [35].

Blood Levels	Normal Diet	Ketogenic Diet	Diabetic Ketoacidosis
Glucose (mg/dL)	80–120	65–80	>300
Insulin (µU/L)	6–23	6.6–9.4	≈0
KB conc (mmol/L)	0.1	7/8	>25
pH	7.4	7.4	<7.3

During physiological ketosis ketonemia reaches maximum levels of 7/8 mmol/L with no change in pH while in uncontrolled diabetic ketoacidosis it can exceed 20 mmol/L with a concomitant lowering of blood pH [11] (Table 1). Blood levels of ketone bodies in healthy people do not exceed 8 mmol/L precisely because the central nervous system (CNS) efficiently uses these molecules for energy in place of glucose.

## 4. Do Ketogenic Diet Work?

The main questions that could be raised are:

(1) Do ketogenic diets works?
(2) Is there a yo-yo effect?
(3) Is a KD safe for obese subjects?

There is no doubt that there is strong supportive evidence that the use of ketogenic diets in weight loss therapy is effective [13], however the mechanisms underlying the effects of KDs on weight loss is still a subject of debate. Atkins’ original hypothesis suggested that weight loss was induced by losing energy through excretion of ketone bodies [36], but more recently different hypotheses have been proposed: one hypothesis is that the use of energy from protein in KD is an “expensive” process for the body and so can lead to a “waste of calories” and therefore increased weight loss compared to other “less expensive” diets [37]. During the first phase of a KD 60–65 g of glucose per day are needed by the body, 16% of this is obtained from glycerol whilst the major part is derived via gluconeogenesis from proteins, of either dietary or tissue origin [38]. Gluconeogenesis is an energy-demanding process calculated at approximately 400–600 Kcal/day (due to both endogenous and food source proteins [37]. There is though no direct experimental evidence to support this intriguing hypothesis, on the contrary a recent study reported that there were no changes in resting energy expenditure after a KD [39]. Some authors claim instead that the results obtained with ketogenic diets could be attributed to a reduction in appetite due to higher satiety effect of proteins [38,40] or to some effects on appetite control hormones [41]. Other authors suggest a possible direct appetite suppressant action of the ketone bodies [42] and more in detail by the BHB that it is supposed to act both as a energy/satiety signal (according to Kennedy’s lipostatic theory) and as central satiety signal *per se* [26]. Over the long term the improvement in fat oxidation mirrored by the decrease of RR (respiratory ratio) could explain the fat loss effect of this kind of diet [39]. Hence we can summarize (listed in order of available evidence) the hypothesized mechanisms of KD’s weight loss effects:

(1) Reduction in appetite due to higher satiety effect of proteins [38,40], effects on appetite control hormones [41] and to a possible direct appetite suppressant action of the ketone bodies [42];
(2) Reduction in lipogenesis and increased lipolysis [33,43];
(3) Greater metabolic efficiency in consuming fats highlighted by the reduction in the resting respiratory quotient [39,44,45];
(4) Increased metabolic costs of gluconeogenesis and the thermic effect of proteins [37,46].

## 5. Other Beneficial Effects in Obesity

It could be argued that the ketogenic diet has beneficial effects other than simply fat and weight loss. For example, Davidson and colleagues [47] recently suggested that ketones may protect from cognitive impairment caused by weight gain and obesity. Moreover there is some evidence that ketogenic diets may have positive effects on mood in overweight subjects [48,49]. Even if during the very early phase of a ketogenic diet (the first 4 or 5 days) subjects may sometimes complain of lethargy [50,51] this effect passes rapidly and subjects subsequently report an improved mood [44,48,49].

Although the prevalence of insulin resistance in obesity is not precisely known, it is quite common [52]. Indeed the first demonstration of resistance to insulin stimulation of glucose uptake was obtained in obese subjects [53]. A primary feature of insulin resistance is an impaired ability of muscle cells to take up circulating glucose and also the ability to slow down hepatic glucose output may be compromised. Thus, individuals with insulin resistance have a fundamental problem metabolizing dietary carbohydrate and will divert a greater proportion of dietary carbohydrate to the liver where much of it is converted to fat (*i.e.*, *de novo* lipogenesis), as opposed to being oxidized for energy in skeletal muscle. Hence the beneficial effects of very low carbohydrate diets in obese subjects are not just a function of weight loss *per se* but also improved glycaemic control, haemoglobin A1c, and lipid markers, as well as reduced use or withdrawal of insulin and other medications in many cases, occurs before significant weight loss occurs. Moreover, in isocaloric experiments, individuals with insulin resistance showed dramatically improved markers of metabolic syndrome than diets lower in fat [54]. 

Another beneficial effect that has been postulated for is related to longevity. Even though available data are restricted to animal models it has been shown that a KD increases AMPK in mice, inhibiting the mTOR/AKT pathway [55]. A ketogenic diet also lowers the serum ratio of IGF/IGF-binding protein 3 in mice with positive effects on metabolic syndrome and cancer risk [56]. In other studies it was reported that a KD increases expression of PGC1-α, a master mitochondria metabolic regulator (that can exerts its therapeutic benefits by increasing mitochondrial biogenesis) [57] and finally it has recently been suggested that β-hydroxybutyrate may act as an inhibitor of class I histone deacetylases (HDACs), a family of proteins that suppress gene expression by deacetylating lysine residues on histone and histone proteins. HDAC inhibitors inhibit tumor growth and have been used to treat several metabolic [58] and neurological diseases [59], moreover HDAC inhibitors can extend the lifespan in *Drosophila* models [60]. It should be stressed that the KD induces a particular metabolic condition that activates fasting pathways during a high or normal energy state and it can also be argued that the transcription of autophagy related genes (fundamental for the anabolic/catabolic equilibrium and hence for whole muscle health) can be activated by KD, mediated by FoxO3 [61,62,63].

## 6. Is There a Yo-Yo Effect?

While there are many studies which demonstrate that a ketogenic diet, at least in the short-term, results in greater weight loss than low-fat diets [13], from a long term perspective the success of a nutritional approach is defined by the amount of weight regain [64]. From this point of view, fewer data are available [41], in particular regarding so-called weight cycling or yo-yo effect [65,66]. Some opponents and doubters of KDs suggest that any beneficial effects are only transient. There is no universally accepted definition of “successful weight loss maintenance” following a diet but a reasonable candidate would be that proposed by Wing and Hill in 2001, which defines it as “individuals who have intentionally lost at least 10% of their body weight and kept it off at least one year” [67]. The criterion of 10% is chosen for its well documented effects in the improvements in risk factors for diabetes and cardiovascular disease, while the 1-yr duration criterion was proposed in agreement with the U.S. Institute of Medicine (IOM) [68]. Recently Sumitharn and colleagues have demonstrated that the increases in circulating ghrelin and in subjective appetite which accompanied a hypocaloric diet were reduced with a ketogenic approach [41]. Moreover we have very recently demonstrated that two brief periods of ketogenic diet separated by longer periods of maintenance of Mediterranean diet, led to successful long term weight loss and improvements in health risk factors without weight regain effect [69].

## 7. It Is Safe for Obese Subjects?

Questions are often raised by colleagues when discussing KDs and a main concern regards blood lipids. In common opinion a low carbohydrate, high protein and fat diet is potentially unhealthy as it may cause a rise in LDL cholesterol and TGs and this issue is of special importance in obese individuals. There are nevertheless several lines of evidence that point to beneficial effects of KDs on these cardiovascular risk factors. The majority of recent studies seem to amply demonstrate that the reduction of carbohydrates can actually lead to significant benefits in total cholesterol reduction, increases in HDL and reduction of blood triglycerides [11,13]. Furthermore KDs have been reported to increase the size and volume of LDL-C particles [70] which is considered to reduce cardiovascular disease risk since smaller LDL particles have a higher atherogenicity. There is a biochemical rational behind the effects of KDs on endogenous cholesterol synthesis. A key enyzme in cholesterol biosynthesis is HMGCoA reductase (the target for statins) which is activated by insulin, which means that an increase in blood glucose and consequently of insulin levels will lead to increased endogenous cholesterol synthesis. Thus a reduction in dietary carbohydrate together with a correct intake of cholesterol will lead to a inhibition of cholesterol biosynthesis.

Another concern regards potential negative renal effects. It is suggested that high levels of nitrogen excretion during protein metabolism can cause an increase in glomerular pressure and hyper-filtration [38]. In subjects with intact renal function higher dietary protein levels have caused some functional and morphological adaptations but without negative effects [71]. It is important though to take into account the renal related effects on blood pressure. The amino acids involved in gluconeogenesis and/or production of urea have, in general, blood pressure lowering effects, while acidifying amino acids tends to cause a rise in blood pressure. Subjects with renal insufficiency, even sub-clinical, kidney transplant patients and individuals with metabolic syndrome or other obesity related conditions will be more susceptible to the hypertensive effect of amino acids, especially of the sulphated variety [72]. The well-documented correlation between obesity and reduced nephron quantity on raised blood pressure puts obese subjects, or those with metabolic syndrome, at risk even if in diabetics with kidney damage the effects are not always consistent with the hypothesis [73,74]. In fact although some authors have reported a positive influence of a reduction in protein intake from 1.2 g/kg to 0.9 g/kg, over the short term, on albuminuria in type 2 diabetics [75], the same authors have subsequently stated instead that dietary protein restriction is neither necessary nor useful over the long term [76]. Furthermore some recent studies have demonstrated that VLCKD can even cause a regression of diabetic nephropathy in mice [77]. However, it is not correct to equate a ketogenic diet with a high protein diet, because *the state of the art* KDs are normoproteic thus the daily amount of protein is about 1.2–1.5 g of protein per Kg of body weight [44,78,79]. With regard to possible acidosis during VLCKD since the concentration of ketone bodies never rises above 8 mmol/L this risk is virtually non-existent in subjects with normal insulin function [80]. Regarding the overall effects of ketogenic diet on health there are differences in opinion about the research. Recently, in a recent systematic review based on limited observational studies, Noto and colleagues suggested a possible harmful effect of low carbohydrate/high protein diet (LC/HP) on health: *i.e.*, an increase of all-cause mortality risk whilst there was no effect on CVD mortality [81]. On the other hand, for example, a large European study demonstrated that an increase in protein content and a reduction in the glycaemic index led to better maintenance of weight loss without differences regarding adverse effects [82]. The existing contradictory evidences on this matter lies in the complex interactions between low-carbohydrate diets and long-term outcomes. Moreover it is important to underline again that a ketogenic diet is not, strictly speaking, a LC/HP; KD is mainly a very low carbohydrate diet with a normal amount of protein that produce a peculiar metabolic state that should not be assimilated to a LC/HP. Some authors claims that a ketogenic diet could affect negatively glucose metabolism [83] but in the cited study the researchers performed the glucose load immediately after the cessation of the KD. It is reasonable to suppose that after a period of very low carbohydrate diet there would be an increased glucose sensitively and for this reason is advisable to have a transition phase from ketogenic diet to a normal diet. Another very recently published study demonstrated that a long-term KD (22 weeks) caused dyslipidemia, a pro-inflammatory state, signs of hepatic steatosis, glucose intolerance and a reduction in beta and alpha cell mass, without weight loss in mice [84]. Two considerations need to be made: the first is that the induction of ketosis and the response to ketosis in man and mouse are quite different and this species metabolic differences could explain why insulin sensitivity is improved in man [54] whilst is decreased in mouse [85] after KD. The second is that 22 weeks is a very long period for a mouse that could be compared to several years in human beings [86,87]. In any case in human subjects the effects of a very prolonged ketogenic diet are, as yet, not well investigated, for this reason, KD may be used safely for a limited period (from 3 weeks to some months) to stimulate fat loss, improve metabolism and help the transition to a correct Mediterranean diet style [69]. Some authors report that a KD induces a severe reduction of IGF-I concentration in rats [88] but, (even if we can compare rat with man) but as stated above a blunting of the IGF-1/AKT/mTOR pathway is not necessary a harmful effect.

Another point that must be addressed is the effect of KD on bone metabolism. There is evidence showing that short-term KDs impair (suggested to be mediated by reductions in circulating IGF-1) bone mass density and mechanical properties of bone in mouse [89]; whilst in humans very long term KD in children with intractable epilepsy may lead to a progressive reduction of bone mineral content [90]. The “caveat” about KD and bone is indispensable but we have to take into account that the study of Bielohuby was performed on growing rats for 4 weeks (*i.e.*, a very long time for humans) and thus we have to consider the above mentioned difference between ketosis in the two species, whilst the condition of a life-long ketogenic diet in intractable epilepsy is beyond the scope of this review.

On the other side, it has been suggested recently that the metabolic complications of obesity, such as type 2 diabetes, metabolic syndrome, impaired glucose tolerance, insulin resistance, hyperglycemia, and inflammation may be associated with an increased risk of fracture and poor bone health. Moreover the amount of visceral fat (related to low-grade chronic inflammation) is associated with lower bone mineral density [91]. Whilst there are many studies on the effects of dietary protein levels on bone metabolism in humans the few investigations on ketogenic diet and bone metabolism were performed on epileptic children. But even if we assume a higher protein intake during a KD (and it is not completely correct as stated above) recently published articles suggested that there is not a negative effect on bone health [92,93,94,95].

## 8. Conclusions

A period of low carbohydrate ketogenic diet may help to control hunger and may improve fat oxidative metabolism and therefore reduce body weight. Furthermore new kinds of ketogenic diets using meals that mimic carbohydrate rich foods could improve the compliance to the diet [78]. Attention should be paid to patient’s renal function and to the transition phase from ketogenic diet to a normal diet that should be gradual and well controlled [69]. The duration of ketogenic diet may range from a minimum (to induce the physiological ketosis) of 2–3 weeks to a maximum (following a general precautionary principle) of many months (6–12 months). Correctly understood the ketogenic diet can be a useful tool to treat obesity in the hands of the physician.
